# “What other option did I have?”– The effect of conflict and displacement on child marriage and early childbearing among displaced Rohingya adolescents

**DOI:** 10.1186/s13031-025-00656-2

**Published:** 2025-03-17

**Authors:** Kate Mieth, Tahia Hasan, Adrija Chakrabarty, Kenna Lee, Adrita Kaiser, Tanvir Hasan, Shatha Elnakib, Caitlin Jackson, W. Courtland Robinson, Linnea A. Zimmerman

**Affiliations:** 1 Johns Hopkins Bloomberg School of Public Health, 615 N Wolfe St, Baltimore, MD 21205 USA; 2 BRAC James P Grant School of Public Health, 28, 6th Floor, Medona Tower, Bir Uttam AK Khandakar Rd, Dhaka, 1213 Bangladesh

**Keywords:** Family formation, Early marriage, Child marriage, Childbearing, Fertility, Fecundity, Humanitarian settings, Cox’s Bazar, Rohingya, Forcibly-displaced Myanmar nationals, Conflict, Displacement, Adolescent health

## Abstract

**Background:**

Humanitarian emergencies are postulated to increase rates of early marriage and early childbearing, as drivers of both are heightened or exacerbated in crisis settings. There is a critical need for research that explores the causal mechanisms that motivate family formation, i.e. the process from marriage into childbearing, and how this process is affected by conflict and displacement.

**Objective:**

This paper aims to describe how displacement and living within a camp context has affected norms and drivers around family formation, focusing on the lived experience of female and male adolescents and young adults.

**Methods:**

We coded and analyzed qualitative data from forty-nine in-depth interviews and sixteen focus group discussions conducted with Forcibly Displaced Myanmar Nationals aged 15–24 who arrived in Cox’s Bazar during or after October 2016.

**Results:**

Participants largely agreed that rates of child marriage have increased post-conflict and displacement. They attributed this increase to a variety of drivers, including fears around protection, socioeconomic need, lack of education and employment opportunities, and a perceived loosening of restrictions around legal age of marriage within camp. While some of these were pre-existing drivers exacerbated by conflict and displacement, others were new drivers that developed as a result. The ways that adolescents and young adults experienced each driver were highly gendered. Conversely, conflict and displacement had seemingly little effect on cultural expectations to demonstrate fecundity immediately after marriage. Finally, participants felt that adverse living conditions within camp have significantly lowered fertility intentions and have increased cultural acceptance and adoption of family planning.

**Conclusions:**

Our results demonstrate that many Rohingya families view child marriage as a practical tool to overcome challenges associated with being displaced, and early childbearing as an inevitable natural consequence of child marriage. The Government of Bangladesh must ease restrictions on income-generating opportunities as well as continue working with humanitarian organizations to provide and fund education and skills-building opportunities for both adolescent girls and boys, who otherwise have no alternatives to child marriage and few other opportunities to productively contribute to their communities.

## Background

Despite the existence of global agreements and laws intended to prevent child marriage, defined as a formal or informal union before the age of 18, approximately 19% of females globally are married before age 18, with 4% married before age 15 [1–4]. Adverse consequences associated with child marriage include early pregnancy, increased odds of intimate partner violence, restrictions on autonomous mobility, social isolation, and discontinuation of education [4–6]. Approximately 3% of males are also married before age 18 globally [4, 7]. Though there have been fewer studies that identify the adverse consequences of child marriage for males, there is evidence to suggest it is associated with lower levels of educational attainment, increased economic pressure, fewer career opportunities, and less awareness on sexual health topics [4, 8].

Child marriage among females has consistently been associated with subsequent adolescent childbearing [6, 8–11]. External pressures for immediate childbearing, including within the family, normalization of risks associated with childbearing, and an increase in social status associated with having children all drive early childbearing [5, 12, 13]. Adolescent girls’ physical immaturity and insufficient nutrition lead to an increased risk of mortality and morbidity for adolescent mothers and their children [5, 12, 14, 15]. Early childbearing is also associated with higher lifetime fertility, again increasing lifetime risk for maternal morbidity and mortality [6].

Though limited and somewhat mixed, current research suggests that rates of child marriage may increase in humanitarian emergencies, as known drivers– including economic uncertainty, lack of employment opportunities, and fear of sexual violence against unmarried women– are often exacerbated in crisis settings [8, 16–26]. Child marriage is also perceived to confer certain benefits, such as increased economic stability and physical protection from violence. Despite the negative health and gender-related consequences of child marriage, it is often used as a coping mechanism to overcome adverse secondary effects of conflict and displacement [21, 22, 27]. However, humanitarian crises have not been universally associated with increases in child marriage [17, 20, 28, 29]. To develop effective strategies to prevent and reduce child marriage within crisis settings, it is critical to understand the preexisting norms and drivers of child marriage within each context and how displacement may affect these drivers.

Research on the direct links between child marriage and early childbearing in humanitarian emergencies is even more limited [29]. Some quantitative research has shown that exposure to conflict can affect fertility behavior [30–32], though the mechanisms by which conflict can affect determinants of childbearing has been largely unexplored [29]. Qualitative evidence among displaced refugees has found that some women express the desire to limit childbearing due to generalized uncertainty about the future [33], while others retain high fertility intentions regardless of displacement [34, 35]. These studies are non-specific to adolescents, however, and largely investigate changes in overall desired number of children, leaving questions about how humanitarian emergencies affect childbearing upon marriage unexplored.

One population that has long suffered the primary and secondary effects of conflict and displacement is the Rohingya. Since being deemed “stateless” in 1982 by their home country of Myanmar (formerly Burma), the Rohingya have endured discrimination, religious persecution, and violence. This has led to decades of mass displacement as Rohingya families have sought asylum in the Muslim-majority country of Bangladesh. Resultingly, Cox’s Bazar, Bangladesh now houses the largest refugee camp in the world [36].

In October 2017, violence towards the Rohingya by the Myanmar government heightened and over 700,000 Rohingya fled to Cox’s Bazar throughout 2017. Rohingya who fled during this time have not been granted refugee status by the Bangladeshi government and are instead referred to as “Forcibly Displaced Myanmar Nationals” (FDMN) [36, 37]. Recent data indicate that this wave of displacement in 2017 may have increased child marriage amongst the Rohingya; approximately 14% of FDMN females age 18–19 and 9.4% of FDMN males age 18–19 reported being married before age 18, relative to 13.3% of registered Rohingya refugee females and 0% of registered refugee males of the same age [38].

Previous studies have shown that child marriage amongst the Rohingya is driven by factors often exacerbated by crisis and camp settings, such as physical and economic insecurity, and pre-existing religious and cultural norms [39–41]. As in other settings, marriage has been viewed as a protective measure for girls that provides them with increased security. Despite general awareness that child marriage is punishable by law in Bangladesh, there is evidence to suggest that the Rohingya perceive the enforcement of child marriage laws to be looser in Bangladesh relative to Myanmar, leading to higher potential for child marriage within the camps [39–41].

Childbearing norms and drivers are less studied among the Rohingya, but some evidence suggests that early childbearing is common. One study within Rohingya refugee camps found that 53.3% of females who married before age 18 had at least one child, pointing to drivers such as increased practice of child marriage, taboos around sexual relations outside of marriage, and perceived loosening of child marriage laws [40]. Yet, very little qualitative research has explored early childbearing among FDMNs specifically. Even less research has been done to understand the childbearing desires and behaviors of Rohingya males, despite widespread acknowledgement that male childbearing preferences and desires influence that of their female partner [42–44].

Given the complexity of the underlying drivers that affect both marriage patterns and childbearing within marriage, there is a critical need for research that explores the causal mechanisms that motivate family formation (i.e. the process from marriage into childbearing) and how this process is affected by conflict and displacement. This paper, which accompanies a quantitative paper [45] that establishes how rates of marriage and childbearing have changed amongst Rohingya FDMN adolescents and young adults, aims to identify and describe how displacement and living within a camp context has affected norms and drivers around child marriage and early childbearing, focusing primarily on the lived experience of both females and males aged 15–24.

## Methods

A mixed methods study was conducted from 2021 to 2023 in Cox’s Bazar, Bangladesh. The quantitative component was conducted by Green Hill: Community Partners International (CPI) and the qualitative component was conducted by BRAC James P Grant School of Public Health (BRAC JPGSPH) with support from CPI. Both components were supported by faculty from the Johns Hopkins Bloomberg School of Public Health, Center for Humanitarian Health.

### Study setting

Cox’s Bazar houses nearly one million displaced Rohingya people, of whom approximately one-third are FDMN [36]. Qualitative research was conducted in camps 1 W, 4, and 17 of Ukhiya Upazila of Cox’s Bazar district. These camps were purposely chosen due to CPI’s previously-established community ties there. The broader camp context is characterized by cramped living conditions, movement restrictions, and limited access to educational and employment opportunities [46–49].

### Data collection

Qualitative data was collected in March-April 2023. Initial participants were identified with the help of CPI, who recommend potential households where adolescents and young adults were likely present. Members of the BRAC JPGSPH research team approached these households, confirmed eligibility, administered informed consent, and conducted initial interviews with those that expressed interest in participating in the study. Remaining participants were identified and recruited via a snowball sampling method. Forty-nine in-depth interviews (IDIs) and sixteen focus group discussions (FGDs) were conducted with FDMNs aged 15–24 who arrived during or after October 2016.

IDIs were conducted using a semi-structured interview guide and included a “Life History and Timeline” participatory method, in which participants were asked to outline what they considered to be the major events in their life from birth to present day. FGDs were conducted using a semi-structured interview guide and included a “Venn Diagramming” participatory method, in which participants were asked to rank how influential different individuals, community contexts, and local health programming are in decision-making around a variety of sexual and reproductive topics. Both the IDIs and FGDs broadly focused on the topics of marriage, childbearing, and family planning. The primary difference between the two methods was scope; the IDIs focused on the participant’s personal experience and opinions on an individual level, whereas the FGDs focused on community-level beliefs and norms.

Considering cultural norms and restrictions around movement amongst Rohingya adolescent girls, both the IDIs and FGDs for female participants were conducted within the participant’s own residence or another participant’s residence. Researchers were instructed to conduct interviews in the most private part of the house and ask that family members allow for privacy. Two data collectors were present at the time of each interview. The data collector who was not directly conducting the interview monitored for other family members during the interviews and engaged them in discussions unrelated to the study to avoid interruption. When a private space within the home was not available, interviews were conducted in an area immediately outside of the home. Male IDIs were conducted at the participant’s home or at a nearby tea shop (at the participant’s discretion) and FGDs were conducted either at one of the FGD participant’s homes or at a CPI-provided training facility.

Interviews with male participants were conducted by male Rohingya volunteers associated with CPI. Due to limited availability of female Rohingya volunteers, interviews with female participants were conducted by female volunteers from the Bangladeshi host community who were fluent in Rohingya and residents of Cox’s Bazar. All data collectors attended a five-day qualitative training workshop that included comprehensive training in ethical conduct of research. Five field supervisors were present during the data collection process to ensure consistent implementation of the research protocol.

Interview guides were developed in conjunction with community advisory boards composed of Rohingya community leaders. To minimize social acceptability bias, social and cultural norms were taken into consideration to ensure that phrasing remained neutral. Interview guides were also pre-tested in the field and revised for clarity and thoroughness per participant and trainee feedback as part of the training workshop. As Rohingya and English are the primary languages spoken by Rohingya volunteers working with local humanitarian aid organizations in camp, the interview guides were provided in both “Rohinglish” (a phonetic spelling of the Rohingya language using the English alphabet) and English, so that the data collectors were able to use both languages to conduct the interview as needed.

Data collectors obtained verbal consent using a pre-written consent script from participants aged 18 years of age or older and married emancipated^1^ participants under the age of 18 prior to conducting each interview. For participants aged 17 and younger, both verbal assent from the participant themself and verbal consent from either their parent or the eldest household member responsible for the participant were collected prior to each interview.

#### Ethical approval

was provided by both The Johns Hopkins Bloomberg School of Public Health Institutional Review Board (FWA00000287) and BRAC James P Grant School of Public Health, BRAC University’s review board (IRB protocol no.: IRB-25 June’22–022).

### Data analysis

English-language transcripts along with observational notes were reviewed, coded, and analyzed using Dedoose 9.0.107. Researchers at Johns Hopkins University and BRAC JPGSPH independently read English-language interview transcripts, then discussed and developed a codebook using a blended coding strategy. The codebook was primarily composed of deductive codes informed by research themes identified during a previous research project conducted in Cox’s Bazar by members of this research team [28]. Researchers also cooperatively added inductive codes to capture emergent themes that did not otherwise fit within the initially-developed codebook.

Researchers met weekly throughout the coding process and engaged in regular memo-writing and reflexivity exercises to both minimize bias and to identify any issues or inconsistencies in code application amongst team members. Once the codebook was finalized and the remaining qualitative data were coded, researchers cooperatively identified emerging patterns and themes across the data, paying special attention to the differences and similarities in the findings that emerged among participant subgroups such as sex, marital status, age at marriage, and age at first birth.

## Results

### Norms and trends around age at first marriage

The reported “ideal” age at first marriage was not entirely consistent with the reported typical age at first marriage within the community. There was some heterogeneity in views around the impact of displacement on rates of child marriage, though most participants felt that child marriage had increased in camp.

For girls, the “ideal” age of marriage was most often reported to be 18 exactly, whereas for boys the “ideal” age generally ranged from 18 to 22. Notably, no participants said that marriage before age 18 was ideal. Most often, they attributed these age preferences to protecting the physical health of women (i.e. to prevent childbearing prior to age 18) and/or to ensuring that adolescents were old enough to be “ready” for the financial (in the case of males), emotional, and social responsibilities associated with married life.

*“If a couple gets married at age 18 or later*,* they will face no difficulties while having children. They will have proper peace in the house and will be able to handle everything. If a girl is married off before the age of 18*,* she will not have enough maturity to handle her emotions and to treat her husband and in-laws the right way […] That’s why it is good to marry off girls after they turn 18.”–unmarried male FGD*,* age 15–24*.

Nonetheless, many participants shared that marriage before age 18 is common. They noted that not all community members view the consequences of child marriage as negative, nor wait until adolescents turn 18 to encourage them to marry.

*“Some people say a girl would be too young to understand how marriage works [before age 18]. […] Others don’t see early marriage as a problem. They believe a girl can learn how to maintain a family after marriage.”–female IDI*,* married at 17*,* age 18*.

A few participants also mentioned community stigma around being an ‘older’ bride, though this stigma did not seem to apply to ‘older’ grooms:

*“If girls get married at an older age*,* people say [bad things about the fact that] they are older*,* but if the boys get married at an older age*,* they do not say anything.”–female* IDI, unmarried, age 21.

At the individual level, many participants discussed the impact that conflict and displacement had had on their own marriage and marriage timing. Most married participants felt that they would have been married later had they remained in Myanmar, the majority of which were themselves married prior to age 18. This finding was consistent across genders. Fewer participants said that they would have gotten married at the same age or earlier if they had stayed in Myanmar, or that they didn’t know. Of those who said they did not know, most felt that their marriage timing was “Allah’s wish”, implying they either didn’t have much personal control in the decision-making around marriage and/or it is not worthwhile to speculate because their life had, by-design, already gone according to “God’s plan”.

At the community level, most (though not all) participants felt that child marriage was more common in the camp as compared to in Myanmar and attributed this rise to a variety of displacement-related drivers, discussed below.

### Child marriage drivers

The most common child marriage drivers participants noted were protection from sexual, physical, and/or social harm, socioeconomic need, lack of educational and/or livelihood opportunities, and a perceived lessening of enforcements around minimum age requirements for marriage in Bangladesh as compared to Myanmar. While these themes were discussed by both male and female participants, the way that they were experienced and applied to adolescents and young adults was highly gendered.

***Protection from Harassment***,*** Assault***,*** and Dishonor***. The threat of sexual harassment and assault was seen as one of, if not the greatest, threat to females’ safety in the camp, eclipsing other related concerns such as maternal mortality or morbidity.

*“We lived in a small place with men all around us*,* so there was a security problem. Thus*,* we decided to marry [my sister] off because we were scared for her safety and security […] We didn’t think about death at childbirth because it was a matter for a later time*,* while the fear of safety and security was already present. So*,* we married her off [at age 17].”– male IDI*,* married at 18*,* age 24*.

The vast majority of participants found living conditions to be both unsafe and unreliable within the camp, particularly for single girls. Getting married was seen as an answer to safety concerns associated with falling victim to eve-teasing^2^ and other forms of sexual harassment commonly faced by young women in camp. This was because married girls were perceived as being more protected than unmarried girls from such types of harassment. There were also fears that verbal harassment could escalate to physical sexual assault, further exacerbating the need to use marriage as a protective strategy.

*“My parents were concerned about my security as I was young and pretty. They feared something might happen to me. Getting me married was a way to ensure my safety. But I don’t know how [that would be true]. Something bad could have happened even if I was with my husband. But that gave them peace*,* so I have nothing else to say on that.”–female IDI*,* married at 16*,* age 22*.

Some participants said that a lack of safety in their home villages in Myanmar similarly drove decision-making around child marriage prior to displacement. However, the threat of sexual violence in Myanmar was described to be less-so from other community members and more a threat from outsiders assaulting their villages. Despite this, many participants felt that protection concerns for girls had noticeably increased since being displaced due to the cramped and dangerous camp setting.

Participants frequently described how the close-quarters conditions of the camps also put an increasing number of unmarried adolescents in close proximity to each other, increasing the likelihood that they would start “illicit” romances or affairs. These “affairs” were often described as a driver of child marriage, as parents chose to marry their adolescent early to prevent them from entering into or continuing to engage in pre-marital relationships that could bring shame or stigma onto themselves and their families. This included culturally inappropriate social contact, pre-marital sex, and unintended pregnancy.

For boys, protection concerns in the camp setting more often had to do with protecting them from the temptation to engage in activities such as harassing women or premarital sexual relationships, rather than protection from physical or sexual harm directed at them. In some cases, fear of single boys being more likely to engage in gang activity and/or in drug use within camp was also mentioned as an impetus for child marriage.

*“Boys can be free from committing sins if they marry earlier. They won’t get involved in a love relationship or get physical with any girl if they are married off at an early age. That’s the practice here.”– male IDI*,* married at age 14*,* age 20*.

***Socioeconomic Protection.*** Participants frequently explained that being displaced had increasingly strained their family’s finances and living situations. For adolescent girls, marriage was a way to alleviate the physical constraints of her family’s tight living quarters and/or to lessen the family’s living expenses, as brides typically move into the groom’s family home immediately after marriage.

Participants also discussed how conflict and displacement had put their family members at increased risk of becoming sick or passing away due to poor migratory and living conditions within camp. For girls, this often incentivized her family to marry her early, for fear that there would be no one to financially provide for her in the case of her parents’ illness or death.

For boys, the increased risk of illness or death due to displacement translated to an increased likelihood that they would need to find a wife to “replace” female family members who were no longer able to perform the type of domestic labor traditionally ascribed to women.

*“When I was in Burma […] we had more relatives and more property […] I would not have had to support my family*,* I could have continued with my studies […] After coming [to Bangladesh]*,* my parents became ill […] If I were to stay home to take care of my parents*,* we wouldn’t have [an income] or food to eat […] That’s why I was forced to get married. My wife now takes care of my parents*,* and I work and earn a living.”–male IDI*,* married at 18*,* age 18*.

Conversely, families’ inability to afford dowries was frequently mentioned as a socioeconomic barrier to marriage for girls, especially amongst unmarried female participants. Participants generally agreed that expectations around dowry amount had increased in Bangladesh relative to Myanmar, despite families having comparatively less. However, there was disagreement regarding whether this was universally true or enforced, and the effect (if any) on rates of child marriage.

There was only one male participant who explicitly mentioned his family’s need for income in the form of a dowry as a clear motivator in his decision to get married:

*“[My bride] was only 13 years old. But I had nothing else that I could do [to support our financial needs]. I had to marry for the sake of my family members.”–male IDI*,* married at age 14*,* age 20*.

### Lack of education and livelihood opportunities

Many male participants expressed great frustration at the lack of educational and career opportunities in camp as compared to those in Myanmar. They believed this to be a notable driver of early marriage within camp. They communicated that the rules which prevent FDMNs from engaging in formal employment opportunities and a lack of formal schooling within camp enticed parents to encourage their adolescent children to get married, as there were few other options to occupy their time and to contribute to the community. Participants also felt the resulting idleness had put more boys at-risk of engaging in eve-teasing, gang activity, and other inappropriate behavior.

*“It would be good if there were a factory*,* as there are so many jobless young boys. These boys tease the girls and sit idly here and there. That [harassment] would stop if they could get work […] Boys didn’t get the chance to do these things [in Burma]*,* as they were busy with their businesses.”–male IDI*,* unmarried*,* age 20*.

Yet even the few male participants that were able to find income-generating opportunities, despite the restrictions and access issues, were often not able to overcome other drivers of child marriage. For example, their having to work left a gap in caretaking and household responsibilities, thus tying back into seeking a wife to fulfill those household roles in their absence.

*“In 2017*,* we came to Bangladesh from Myanmar. We had nothing to do [upon arriving] […] I wanted to study*,* but I couldn’t. My father told me to work in his shop […but] someone had to take care of the household chores*,* take care of my parents*,* maintain the house*,* and do everything. My parents cannot do that anymore. Now I am in the shop the whole day. What other option did I have [but to find a wife to do those tasks]?”–male IDI*,* married at 18*,* age 19*.

By contrast, girls were restricted from education during much of their adolescent years even in Myanmar due to cultural norms. Consequently, very few female participants expressed a similar frustration with the lack of educational and livelihood opportunities. However, girls being forced to stay within their increasingly cramped houses for such a significant portion of their adolescence with “little else to do” was occasionally described as another driver of early marriage for girls.

### Limited enforcement of minimum age requirement for marriage

Participants often directly compared the restrictions around marriage prior to age 18 in Myanmar to those in Bangladesh, but there was not clear consensus regarding whether participants perceived restrictions as being more, less, or similarly strict to those in Bangladesh.

Participants that thought child marriages were more common post-displacement most often felt that marriage restrictions were looser and/or there was a relative disregard for child marriage.

laws in Bangladesh as compared to those that regulated child marriage in Myanmar. Several female participants who were married prior to age 18 admitted that they or their family purposefully lied about their age, forged documents, and/or did not notify the camp in-charge (CIC) of their marriage until they were age 18 to avoid penalization or repercussions.

*“I was 13 when I got married […] [my mother and in-laws] arranged a fake birth certificate for my marriage and*,* according to this certificate*,* I was 19.”–female IDI*,* married at 13*,* age 15*.

Fewer participants (and more often males) felt that the CIC was able to adequately restrict child marriage within camp. They described a perceived tightening of marriageable age restrictions and their enforcement in camp due to stricter monitoring by camp officials. This was the most common reason given by those who felt child marriage rates had not risen since being displaced.

### Childbearing norms

***Time to First Birth.*** When asked about the effect the conflict and/or displacement has had on childbearing, no participants discussed changes in time to first birth, nor described there being any difference in the pressure to have a child soon after marriage in Bangladesh as compared to Myanmar.

Participants universally described the societal expectation of initiating childbearing shortly after marriage, typically within the first year. These expectations were shared by married women and men, illustrating the strength of the norm around demonstrating fecundity immediately after marriage and resistance to delaying first birth across both genders. The only exception was in certain cases where girls were married very young. However, child marriage did not universally exempt younger brides from fulfilling what was considered their wifely obligation.

*“If you marry at the age of 18*,* you have a child at the age of 18. If you marry at the age of 20*,* you have a child at the age of 20 […] you [always] have a child in the first year of marriage. This is the culture of us Rohingyas. Parents think that if [their married children] are too old*,* they will not be able to raise their children well. Grandparents want to see their grandchildren’s faces.”–married male FGD*,* age 15–24*.

Perceived consequences of delaying first birth included social stigma, not solidifying the bond between husband and wife, marital arguments, and girls specifically being accused of being infertile or “barren”.

*“When I didn’t become pregnant in the 1–2 years after my marriage*,* everyone started saying bad things about me: that I couldn’t have a child*,* that I am a “Baja” (infertile) woman*,* and that my husband should remarry. My neighbors*,* my relatives […] everyone was bad-mouthing me.”–female IDI*,* married at 12*,* age 18*,* 1 child*.

Another perceived consequence was the threat of a husband seeking a second wife (i.e. choosing to enter a polygamous marriage)– the rationale being that a second wife might be able to provide him with a child. Female participants often described choosing to have a child quickly after marriage to keep their husbands invested in the relationship.

*“Most [girls] have a baby quickly [after marriage] […] [because] when you have a child*,* affection is born in the mind of the husband […] so he won’t marry anyone else.”–married female FGD*,* age 15–24*.

Despite this strong societal expectation, several IDI participants did specifically mention wanting to (or wishing that they could) delay childbearing after their marriage. All of these were women, and all but one were married before age 18. There were also several participants, particularly those who participated in the FGDs, who said that it would theoretically be ideal to wait until age 18–20 + to start having children, especially for women. Their reasoning typically was to preserve the health of the mother and to ensure that the couple was “prepared” or “mature enough” to be parents themselves. However, societal expectations around shorter times to first birth almost always took precedence over personal preference.

#### Fertility Rate, Birth Spacing, and Family Planning

By contrast, almost all participants agreed that couples were having far fewer children and using family planning more in camp than they did in Myanmar. They described increased flexibility in making decisions around how many children couples wanted and preferences around birth spacing in Bangladesh relative to Myanmar. They said couples generally do not experience the same level of heavy societal pressure after the birth of their first child and were even encouraged to practice birth spacing in many cases.

*“[My in-laws] don’t want us to have another baby right away. They know how much pain I had to endure during the birth of my first baby at such an early age. So they told us to wait until our first child grows up.”–female IDI*,* married at 17*,* age 19*,* 1 child*.

When asked, participants of both genders typically stated that they wanted to wait 3–6 years between having their children. Family planning was openly discussed to achieve desired birth spacing and use of family planning seemed to be significantly less stigmatized after the birth of a first child. Though a few participants did mention religious stigma around family planning, saying it was considered a sin, participants more often described family planning as being beneficial, particularly in promoting the health of the mother and child.

*“My first child was unplanned. To avoid another unwanted pregnancy*,* I am taking birth control pills. If I conceive another child [now]*,* then none of my kids will get a better life. After my firstborn is all grown up*,* I will consider having another child.”–female IDI*,* married at 19*,* age 20*,* 1 child*.

Many attributed the increased use of family planning to improved access to health education and family planning methods in camp.

*“The rate of using family planning methods has increased here. [In Myanmar] there was just 1 method available*,* which was injections [Depo-Provera]. People didn’t know about other methods*,* or even if others existed. They also didn’t consult with their doctor about family planning methods. Now couples discuss family planning and go to the hospital [to seek it out] and community health volunteers come [to our homes to discuss family planning] frequently.”–unmarried male FGD*,* age 15–24*.

Many participants also cited constraints to childbearing due to living in camps specifically, the most common being a lack of educational opportunities, followed by lack of space for children to live in and/or play, shortage of money/income, and poor access to health services. These sentiments were strongly held and evenly shared by both male and female participants.

***“****Interviewer: Are people having children at the same age here?*

*Participant: Yes. But they could have had more [children] in Burma*,* they are having fewer children here. There is no space to live*,* there is no arrangement for education*,* and there is no food. In short*,* you can’t feed*,* you can’t teach*,* and there’s no income - that’s why people are having fewer children [in Bangladesh].”–married female FGD*,* age 15–24*.

## Discussion

Displacement and living within a camp context have amplified some previous drivers of child marriage amongst the Rohingya. This is especially true of more practical drivers, such as concerns of sexual violence and the socioeconomic incentives surrounding marriage. In other cases, displacement and living within a camp context has introduced new drivers, such as concerns of increased illness and mortality among family members and severely limited education and livelihood opportunities (the latter for boys especially). However, there appeared to be no impact on norms around girls getting married younger than boys, nor the pace of the transition from marriage into parenthood. Displacement did appear to strongly reduce the total number of desired children due to severe living conditions within camp and increase use of birth control.

We found consensus that the ‘ideal’ age of first marriage for girls was 18 exactly and was slightly older for boys. The consistent and explicit mention of age 18 likely points to strong messaging from either the CIC and/or local NGOs reinforcing that 18 is the minimum legal age of marriage. While our findings echo another study [40] that similarly found age 18 to be the preferred minimum marriageable age, two previous studies [39, 41] found a stronger preference for marrying prior to age 18 amongst community members. It is unclear if social desirability bias may partially explain our findings, or if participants have genuinely adopted the messaging surrounding “ideal” minimum age for marriage in recent years.

Despite wide-spread agreement of an “ideal” age, both our qualitative and quantitative results demonstrated that child marriage remains common and accepted, particularly for girls. Our quantitative research [45] found a sharp increase in risk of marriage at exactly age 18 for girls. These combined findings point to a practice within the community of getting married upon turning 18 for girls or, perhaps, a tendency to report a marriage only once the bride and/or groom have turned 18. Our quantitative results also mirrored our qualitative finding that girls tend to marry younger than boys. This is likely at least partially due to the cultural stigma put on “older” brides that does not exist in the same way for boys, which was similarly demonstrated in previous research [39–41].

Participants largely reported that displacement and living within camp has led to an increase in child marriage within the community and most married participants felt that they would have married later had they stayed in Myanmar. Interestingly, this perceived increase in child marriage rates is seemingly at odds with our quantitative findings, which showed lower rates of child marriage amongst the younger cohort (aged 15–19) as compared to the older cohort (aged 20–24) [45]. There are two possible explanations for this discrepancy. The first could be reporting bias; recently-married adolescents younger than age 18 may have been less likely to report their early marriage for fear of legal repercussions, leading to an undercount of the actual rate. Another explanation could be the difference in reference periods between quantitative and qualitative methods. Our quantitative findings compared the rate of child marriage between younger and older age cohorts, whereas our qualitative research asked participants to compare rates of child marriage within camp to the rate of child marriage in Myanmar *before* the latest wave of violence in 2017, which is a broader timespan.

Overall, our qualitative findings indicate that child marriage appears to largely serve as a tool to improve uncertain and vulnerable situations for Rohingya adolescents, young adults, and their families, rather than an ideal or spiritually superior situation by which to form a family. The rationales by which this ‘tool’ was employed, that is, the motivations why a girl or boy would be married early, were highly gendered and often directly related to living within a camp setting.

For girls, concerns of sexual, physical, and/or social harm were paramount. This fear was particularly salient in the camp setting, where conditions were generally considered more unsafe, especially for unmarried adolescent girls. This is consistent with other literature [39, 41] that highlighted the role of marriage as a tool for protection from physical and sexual violence amongst the Rohingya– both pre- and post-displacement.

A unique finding from our research was that marriage was also viewed as a tool to protect boys from perpetrating the types of immoral acts (e.g. eve teasing) and/or violence that were seemingly more common in the camp setting. Thus, child marriage for boys was a way to both dissuade “bad” behavior that would otherwise dishonor themselves and their families and to secure more respected status as a husband within their community.

For both genders, marriage was also a way to rectify premarital relationships that formed against parental will, especially as close-quarters camp conditions were believed to put adolescents at increased risk of such relationships. Similar themes around using marriage as a tool to preserve family honor and premarital purity were described by several other studies conducted with Rohingya adolescents [39–41], underpinning the strength and consistency of this driver over time.

Marriage also served as a major socioeconomic tool, again presenting in highly gendered ways. Consistent with other literature [39], girls served as a form of socioeconomic currency, benefitting both the household of origin (by reducing the family’s living expenses and increasing space within cramped living conditions) and their new husband’s home (by providing unpaid household labor and often a dowry). Patriarchal gender norms regarding labor played a large factor in this marriage economy. Females were expected to take on traditional household and caretaking duties, whereas men were expected to act as the traditional breadwinners.

Increased risk of illness and death due to conflict and displacement seemed to upset this economy in two ways– girls’ parents increasingly married their daughters early for fear of their being left unprovided for, and boys and their families increasingly sought out brides to provide household labor when other female family members were unable due to illness or death. The latter was seemingly true even when boys were otherwise unoccupied themselves due to the general lack of educational and or livelihood opportunities. Considering the dearth of research that includes male participants, we are limited in our ability to assess consistency with other studies, underscoring the importance of including male perspectives in future research.

Rohingya have long been prohibited by the Bangladeshi government from working due to their FDMN status. Moreover, there was historically no formal system of schooling within the camp and all educational opportunities were provided privately via NGOs or tutors [46, 48–50]. Without these typical outlets, adolescents and young adults were left with few other ways to occupy their time, to demonstrate independence, and to contribute to their community. Thus, marriage may have acted as one of the only remaining milestones of reaching adulthood for FDMN adolescents at the time this research was conducted.

The Bangladesh government has since endorsed and begun implementing a “Framework on Skills Development for Rohingya Refugees/FDMNs and Host Communities” [51]. This framework aims to provide Rohingya with increased skills and capacity-building opportunities commensurate to those available in Myanmar, with the ultimate goal of preparing FDMNs for repatriation to their home country [51, 52]. Resultingly, partners from the Inter-Sector Coordination Group (ISCG)’s Education sector, with support from the Bangladesh government, have opened formal K-10 education centers within camp using a “Myanmar Curriculum.” Additionally, ISCG’s Livelihoods and Skills Development sector (which was newly formed to operationalize the 2022 framework) has rolled out variety of pre-vocational and vocational skills-building opportunities for FDMN and refugee children and adolescents. Still, true income-generating opportunities continue to be in short supply and secondary school enrollment remains particularly limited, especially for girls. The latter is largely due to the same gender inequality issues described here– including families requiring or needing girls to stay home to complete household chores, security concerns, and lack of gender-separated classes [37, 53–56].

The creation of safe spaces for unmarried adolescents to gather may be a useful compliment to the expansion of education and livelihood opportunities. Our participants, both married and unmarried, expressed a desire to have more spaces like our focus group discussions to share with their peers. Though outside the scope of this paper, qualitative data on premarital knowledge did indicate that adolescents and young adults do not often have opportunities to discuss child marriage and childbearing. Due to existing gender norms, separating these groups by gender would likely be critical for community acceptance. This would, however, also give facilitators the chance to discuss the gender-specific ways that the conflict and displacement have affected both girls’ and boys’ experience of child marriage and early childbearing. In this way, they may be able to provide gender-appropriate resources to participants and families looking for “tools” other than child marriage to overcome common challenges that they face in camp.

Other studies identified religion as a notable factor underpinning early marriage and that the teachings of Islam directly mandate earlier marriage, particularly for girls at the onset of menarche, within the Rohingya community [39–41]. We did not find strong evidence of this. While our interview guides did not specifically inquire about the role of religion, open-ended questions about drivers of early marriage rarely produced mention of religion, Islam, or related teachings. Moreover, as part of a participatory Venn diagraming activity, FGD participants were asked to categorize people and events into categories as having the “most influence”, “a lot of influence” or only “some” influence regarding decision-making around marriage age and timing within their community. FGD participants overwhelmingly put religious figures such as Imams and Majhees in the “some influence” category, and others did not mention religious leaders at all. While our findings do not necessarily contradict previous findings around religion, they may point to the increasing importance of practical drivers after experiencing prolonged displacement.

We found less evidence of the impact of displacement on transitions into childbearing upon marriage. There was a strong cultural custom for near-immediate childbearing after marriage, regardless of the couple’s ages. The pressure that young brides specifically face to conceive early is reflected in qualitative data from several non-humanitarian studies [5, 9, 12] as well as in previous studies conducted with Rohingya adolescents specifically [40, 41]. Social pressures to conceive early seem to have been present prior to the conflict according to our participants, indicating that this norm was largely unaffected by the displacement experience. This finding was further reinforced by our quantitative findings, which demonstrated that there was no difference in time to childbirth after marriage between age cohorts or by age at marriage [45].

Other norms around fertility, and particularly those around subsequent childbearing, were strongly affected by displacement and living within a camp setting. Numerous respondents identified the challenges of raising a family within the camp’s limited resources. They reported choosing to practice family planning and to have fewer children in Bangladesh so that they could better provide for the children that they already had. This seems to have opened the door for couples to choose to have fewer children and to practice increased birth spacing, if they so desire. It has also seemingly increased acceptance of and use of family planning, so long as it is *after* the first child is born and family connections have been cemented. However, our finding differs from a previous study conducted with the Rohingya, which reported a strong preference for a large family size and low birth control use [41]. It is possible that social norms around fertility and family planning have notably shifted since the aforementioned study was published in 2018, perhaps due to continued financial constraints and increasing access to sexual and reproductive health services as a result of prolonged displacement.

Current programs geared towards child marriage and early childbearing are primarily, if not exclusively, focused on prevention– which leaves the population of already-married adolescents with very few resources and outlets for support regarding their health and wellness within the marriage. Creating spaces for recently-married adolescents and young adults to gather may provide an opportunity to discuss sexual and reproductive health topics generally considered “taboo” prior to marriage, such as family planning.

Programming about birth spacing, particularly targeted at younger married adolescents, could serve as an entry point to discuss family planning and use of birth control– both of which are gaining acceptance within the community. Adolescent sexual and reproductive health programs that promote delaying first births, however, are unlikely to be successful without broader community engagement to address the pervasive norms surrounding the importance of demonstrating fecundity immediately upon marriage. Practitioners must work with community leaders such as Majhees, who may be more aware of when child marriages occur, to encourage participation in health workshops early on in marriage when couples are just beginning to establish their families.

Our study should be considered in light of several limitations. Due to limited availability of female Rohingya volunteers, only male data collectors were Rohingya, while female data collectors were part of the host community. Despite our including topics such as cultural sensitivity and minimizing bias as part of the data collectors’ training, it is probable that longstanding tension between Rohingya and host communities [50] limited the level of openness our female participants felt with their interviewers, likely affecting the correctness and/or depth of some of the responses. Additionally, stigma around movement for young adults, and particularly unmarried girls, limited the amount of privacy that interviewers had when discussing these sensitive topics in cramped living conditions. While data collectors were trained in how to create as private an area as possible given these physical limitations, it is conceivable that participants modified their responses out of fear that another family member would hear their answers and disapprove. Fear of being overheard was the most common reason adolescents/young adults declined to participate in the study when approached, though refusal to participate happened infrequently.

Despite these limitations, our study has many strengths. We recruited a diverse response pool composed of both married and unmarried males and females to ensure a comparative perspective, both in terms of marriage or gender. Very few other studies have included males in studies of child marriage, despite their own risks for marriage and the influence they have on female partners. Additionally, we partnered with an established organization, that was already well-respected and trusted within the community.

## Conclusion and recommendations

Our participants largely felt that child marriage is on the rise and most child marriage drivers discussed by our participants were practical considerations rather than a deep-set belief in the moral or religious imperative to marry early. Early childbearing seemed to be much less affected by conflict and displacement, but fertility desires seem to have significantly reduced due to resource constraints within camp.

Significant strides have been made in both the education and livelihoods and skills building sectors to restore the types of educational and livelihood opportunities that have been lost due to conflict, displacement, and living in a camp setting since this research was conducted. However, gaps in coverage and access remain, particularly for girls, and especially at the secondary education level. Continuing to fund and restore the types of academic and economic opportunities that are typically available in non-camp settings will provide the best alternative to early marriage as a means of establishing adolescents’ transition into adulthood and preventing ‘idleness’ borne of camp constraints. Increasing complimentary income-generating opportunities for FDMNs will also provide adolescents, young adults, and their families options other than child marriage to financially support their families. Creating gender-specific health education-oriented groups for both unmarried and married adolescents and young adults may additionally help practitioners to meet youth demand for more safe spaces to gather and share experiences around these topics. Finally, addressing many of the restrictive gender norms that promote child marriage and childbearing, as well as limit girls’ participation in the growing number of education and skills-building opportunities in camp, require broader community commitment. Parents and community leaders must be engaged to identify and challenge some of the most pervasive gender norms that contribute to child marriage and early childbearing.

## Data Availability

The qualitative datasets generated and analyzed during the current study are available from the corresponding author on reasonable request.

## Footnotes

1 Individuals married prior to age 18 are automatically considered emancipated minors due to their marital status.

2 “Eve teasing” refers to the act of males making unwanted sexual remarks or advances towards a female in a public space.

## Abbreviations

BRAC: JPGSPH BRAC James P Grant School of Public Health
CIC: Camp-in-Charge
CPI: Community Partners International
FDMN: Forcibly-Displaced Myanmar National
FGD: Focus Group Discussion
IDI: In-depth Interview
ISCG: Inter-Sector Coordination Group
NGO: Non-governmental Organizations
